# Air-Void Stability in Self-Compacting Concrete: Linking Fresh-Air Retention with Hardened Pore Structure Through a Synthetic Dispersion Approach

**DOI:** 10.3390/ma19132730

**Published:** 2026-06-25

**Authors:** Beata Łaźniewska-Piekarczyk, Patrycja Miera, Mateusz Moskal

**Affiliations:** Department of Building Engineering and Building Physics, Faculty of Civil Engineering, Silesian University of Technology, Akademicka 5, 44-100 Gliwice, Poland

**Keywords:** self-compacting concrete, air entrainment, air-void system, spacing factor, micropores, pore structure, fresh-air retention, freeze–thaw resistance, Graeco-Latin square, ANOVA, synthetic dispersion indicators

## Abstract

**Highlights:**

- Fresh-air retention and hardened air-void structure were jointly analysed in 25 SCC mixtures using a five-level Graeco-Latin square design.
- ANOVA distinguished significant effects, near-threshold tendencies, and non-significant trends separately for each response variable.
- Water-to-binder ratio significantly affected early air loss and micropore content, while binder type showed near-threshold tendencies.
- Early air retention measurements after 5 and 15 min strongly correlated with 28-day hardened air content.
- Similar total air contents did not yield equivalent pore-spacing characteristics, requiring joint analysis of multiple air-void descriptors.

**Abstract:**

Air entrainment in self-compacting concrete (SCC) is governed by coupled interactions between chemical admixtures, empirical workability behaviour, aggregate-skeleton geometry and early air-bubble stability. In highly flowable mixtures, the hardened air-void system cannot be assessed reliably from total air content alone because bubble escape, redistribution and coalescence in the fresh state may change the final pore structure. This study evaluates the link between early fresh-air retention and hardened air-void characteristics in 25 SCC mixtures arranged according to a five-level Graeco-Latin square design. The analysed factors were air-entraining admixture (AEA) dosage (0.00–0.20% by mass of cement), binder type, water-to-binder ratio (0.29–0.41) and the volumetric paste-to-aggregate filling parameter φ (1.1–1.5). The aggregate skeleton was kept constant to separate paste-composition and volumetric-filling effects from aggregate grading. Fresh concrete was characterised by slump-flow diameter, *T*_50_ flow time, density and air content after 5 and 15 min; these quantities were treated as empirical workability and early-retention indicators, not as direct rheological parameters. Hardened concrete was examined after 28 days according to EN 480-11 using total hardened air content *A*, spacing factor *L*, micropore content *A*_300_ and specific surface *α*. The slump-flow diameter ranged from 50 to 79 cm, fresh air content after 5 min from 1.6% to 8.6%, air loss between 5 and 15 min from 0.41 to 1.12 percentage points, hardened air content from 1.20% to 8.59%, and spacing factor from 0.13 to 0.44 mm. Strong correlations were obtained between fresh and hardened air contents (*A*_5_ vs. *A*: r = 0.920, R^2^ = 0.846, *p* < 0.001, 95% CI for r: 0.824–0.964; *A*_15_ vs. *A*: r = 0.922, R^2^ = 0.849, *p* < 0.001, 95% CI for r: 0.828–0.965), while hardened air content was strongly and inversely related to spacing factor (*A* vs. *L*: r = −0.907, R^2^ = 0.822, *p* < 0.001, 95% CI for r: −0.958 to −0.797). The recalculated ANOVA showed that statistical significance was response-dependent: w/b was significant for early air loss ΔA (F = 4.190, *p* = 0.040, partial η^2^ = 0.677) and micropore content *A*_300_ (F = 4.058, *p* = 0.044, partial η^2^ = 0.670), whereas binder type showed near-threshold tendencies for fresh and hardened air contents. No single factor was statistically significant for all air-void descriptors. The SDI-based approach is therefore presented as a bounded explanatory framework, not as an externally validated prediction model. Direct durability claims, including freeze–thaw resistance, require separate experimental verification.

## 1. Introduction

Self-compacting concrete (SCC) is designed to flow under its own weight, fill form-work and pass through reinforcement without vibration [1]. This technological advantage is obtained by a high paste volume, optimised aggregate grading and chemical admixtures [2]; however, these same features make SCC particularly sensitive to the stability of entrained air [3]. In air-entrained SCC, the performance of the material depends not only on the total amount of air introduced into the fresh mixture, but also on the ability of this air to remain stable until setting and to form an appropriate hardened air-void system [4]. The stability of air in SCC is more difficult to control than in conventional vibrated concrete [5]. The low yield resistance and high deformability of the fresh mixture may facilitate bubble migration and escape [6], whereas the presence of polycarboxylate ether-based superplasticizers (PCEs) may alter the adsorption behaviour of air-entraining admixtures (AEAs), liquid-film stability and bubble coalescence [7]. Therefore, air entrainment in SCC should be treated as a coupled physicochemical and technological process, rather than as a simple function of AEA dosage [8]. From a durability-oriented perspective, the hardened air-void system is commonly described by total air content, spacing factor, specific surface of air voids and the amount/number of small pores [9]. These parameters are associated with the potential protective role of entrained air; however, they cannot be used as direct proof of freeze–thaw resistance unless freeze–thaw tests are performed [10]. This distinction is important in the present work: this study analyses air-void descriptors and their formation mechanisms, but it does not directly verify freeze–thaw durability [11]. In the fresh state, the final air-void system is shaped by several simultaneous processes [12]. Air bubbles are generated during mixing, stabilised by surfactant action, transported within the cement paste and constrained by the aggregate skeleton [13]. Their survival depends on the balance between buoyancy, apparent resistance to flow, coalescence and geometrical confinement [14]. For this reason, the transition from fresh air content to hardened air-void structure should be analysed using both time-dependent air measurements and hardened pore descriptors [15]. The geometry of the aggregate skeleton is particularly relevant in SCC because the paste phase fills intergranular spaces and controls the mobility of air bubbles [16]. The volumetric filling parameter φ used in the Synthetic Dispersion Indicators (SDI) concept expresses the relationship between the apparent volume of cement paste and the aggregate skeleton [17]. A higher or lower degree of paste filling may change the available space for bubble displacement, coalescence, and stabilisation and may therefore influence the spacing factor independently of the total air content [18]. The SDI concept offers a useful framework for interpreting concrete as a multi-phase dispersion system [19]. In this approach, the cement paste, aggregate skeleton and their volumetric relationship are described using synthetic parameters that can be linked with technological properties [20]. In the present study, this concept is not used to claim a universal predictive model. It is used to organise the experimental interpretation of air entrainment in SCC by combining mixture composition, fresh-air retention and hardened pore structure [21]. Previous studies on air entrainment have generally focused either on conventional concrete, on the total air content of fresh mixtures, or on selected hardened air-void descriptors [22]. This creates a methodological gap for SCC, where the air-void system is shaped by the simultaneous action of high deformability, paste volume, PCE-assisted dispersion, surfactant-controlled bubble stabilisation and aggregate-skeleton confinement [23]. For this reason, a direct transfer of conclusions from conventional vibrated concrete to SCC is not sufficient [24]. The recent literature increasingly emphasises multiscale dispersion, admixture compatibility and pore-structure refinement in cementitious and alkali-activated systems [25]. For example, nano-silica-modified geopolymer studies have shown that dispersion state can govern gel-network uniformity, pore refinement and mechanical response. Although these systems differ from SCC, they support the broader conclusion that the spatial distribution of dispersed phases may be as important as their total volume. This concept is directly relevant to air entrainment, where similar total air contents may produce different spacing factors and micropore fractions [25,26]. The present work therefore does not treat SDI as a universal prediction model. It uses the SDI concept as an interpretative framework for connecting mixture composition, early air retention and hardened pore geometry. This distinction is essential because the reviewers’ concern regarding overinterpretation is scientifically justified: without independent validation, the SDI formulation should support explanation and hypothesis building, not general prediction. The research gap addressed in this paper is the absence of an integrated, statistically transparent interpretation of early air retention and hardened air-void structure in SCC, with explicit separation of statistically significant effects, near-threshold tendencies and mechanistic but non-confirmed trends. This study also addresses the practical problem that total air content alone may be misleading when the pore-spacing factor, micropore content and specific surface are not analysed simultaneously. The aim of this study was to evaluate the influence of AEA dosage, binder type, water-to-binder ratio and the volumetric filling parameter φ on fresh-air retention and hardened air-void structure in SCC. The experimental programme consisted of 25 mixtures arranged according to a five-level Graeco-Latin square design. The response variables included slump-flow diameter and flow time, fresh air content after 5 and 15 min, air loss over time, density of the fresh mixture, total hardened air content, spacing factor, micropore content and specific surface of air voids. The novelty of the work lies in linking early air-retention indicators with hardened air-void descriptors within a controlled Graeco-Latin square design and interpreting these relationships through a bounded SDI-based dispersion framework. This study deliberately limits its conclusions to the investigated materials and mixture range. The proposed interpretation is intended to support mechanistic understanding and to define directions for further validation by direct rheometry, longer air-retention monitoring, image-based bubble analysis and freeze–thaw testing.

## 2. Methodology

### 2.1. Materials

The experimental programme was carried out using SCC mixtures designed to evaluate air entrainment, early fresh-air retention and hardened pore structure under controlled variation of four factors: AEA dosage, binder type, water-to-binder ratio and the volumetric paste-to-aggregate filling parameter *φ*. The materials used in this study are summarised in Table 1. The values reported in Table 1 should be interpreted as material-characterisation or supplier-based design data used for mixture design and discussion. They were not treated as independent variables in the statistical model, and the article does not claim a complete physicochemical characterisation of each binder by XRF, XRD, SEM, FTIR or TGA.

Five binders were used: CEM I with silica fume, CEM II/B-V, CEM II/B-S, CEM II/B-M and CEM III/A (Górażdże Cement SA (Chorula, Opolskie, Poland)). This selection enabled the assessment of mixtures with different mineral additions and surface characteristics. The statistical interpretation was not based on chemical composition alone but on the response of the complete SCC systems presented in Table 2 and Table 3. The aggregate skeleton was kept constant in all mixtures in order to isolate the influence of paste volume and composition.

Natural river aggregate with a maximum grain size of 8 mm was used as the granular phase. The aggregate was washed and sieved to ensure a controlled grading curve and to eliminate the influence of impurities on the air-entrainment process. The packing characteristics of the aggregate were maintained constant throughout the study in order to isolate the effect of volumetric relationships between paste and aggregate. The dispersion index of the aggregate skeleton, expressed in terms of intergranular porosity and specific surface, was determined experimentally and treated as a constant parameter within the SDI framework.

The mixing water used in this study was potable water with no additional chemical treatment. The total water content included both free water and the water introduced with chemical admixtures, ensuring an accurate determination of the water-to-binder ratio.

A polycarboxylate ether-based superplasticizer was used to obtain SCC workability. The dosage of superplasticizer was adjusted during mixture design with the technological target of a slump-flow diameter of approximately 675 mm.

The air-entraining admixture based on surface-active agents was used to introduce and stabilise air bubbles. The correct dosage range was 0.00–0.20% by mass of cement, with five levels: 0%, 0.05%, 0.10%, 0.15% and 0.20% mass of cement. This notation is used consistently in Table 2 and Table 3. The range covers non-entrained, moderately entrained and highly entrained systems and enables the analysis of both air formation and possible air loss over time.

### 2.2. Methods

Fresh concrete was assessed using air content according to EN 12350-7 [29], slump-flow diameter and T_50_ flow time according to EN 12350-8 [30] and density according to EN 12350-6 [31]. Slump-flow diameter and T_50_ time are reported as empirical workability indicators. They are not direct rheological parameters such as yield stress, plastic viscosity, thixotropy or viscoelastic moduli; therefore, all rheology-related interpretations in the Discussion section are deliberately restricted to workability-level observations.

Air-entrainment stability was evaluated by comparing the air content measured after 5 min and after 15 min. This time interval describes early air retention under laboratory conditions and should not be interpreted as a complete simulation of transport, casting or setting. To improve the quantitative interpretation of the data, additional derived indicators were calculated from the measured values in fresh concrete and from the hardened air-void parameters.

The hardened air-void system was analysed after 28 days according to EN 480-11 [32] using the linear-traverse method. The analysed descriptors were total hardened air content *A*, spacing factor *L*, micropore content *A*_300_ and specific surface *α*. The values of porosity parameters of hardened concrete are based on two determinations and are therefore used primarily for comparative interpretation between mixtures, not as a full statistical population for durability classification.

The statistical evaluation was performed using ANOVA within the Graeco-Latin square design. The response variables were analysed separately: air content after 5 min, air content after 15 min, air loss between 5 and 15 min, hardened air content, spacing factor, micropore content and specific surface. The complete ANOVA tables report degrees of freedom, sums of squares, mean squares, F-values, *p*-values and partial η^2^. Pearson correlation coefficients were calculated for the main fresh-to-hardened and pore-structure relationships, and 95% confidence intervals were obtained using Fisher transformation. The design was used as a screening and interpretation tool for main effects; it does not fully resolve physicochemical interactions between PCE, AEA, binder chemistry and paste volume.

#### 2.2.1. The Concept of Synthetic Dispersion Indicators of Concrete

The escape of air bubbles from the concrete mixture depends on the axis of their buoyancy. The resulting number of emerging bubbles depends on the properties of the cement paste and the size of the available inter-grain spaces. Based on the analysis of the test results [1,2,3,4,5,6,7,8,9,10,11,12,13,14,15,16,17,18,19,20,21,22,23,24,25,26,27,28,29,30,31,32,33,34,35,36,37], the most important factors influencing the material’s air-entrainment effect and the resulting porosity structure were selected, i.e., the type of binder, the water-to-binder ratio, the size of inter-grain spaces and the SCC air entrainment degree. In works [12,13,35], the idea of Synthetic Dispersion Indicators (SDI) was proposed to describe the properties of concrete.

The stability of the air-void system in concrete has been a subject of extensive research for decades. Early fundamental studies focused primarily on operational and environmental factors; for instance, Gaynor and Mullarky [38] evaluated the distinct effects of mixing speed on initial air content, while Powers [39] established the baseline theoretical frameworks regarding freezing effects and hydraulic pressure in hardening cement pastes. As concrete technology advanced, the introduction of high-range water reducers altered these dynamics. Kobayashi et al. [40] and Litvan [41] were among the first to systematically investigate the frost resistance and air-entrainment mechanisms of concrete in the presence of superplasticizers, noting both advantages and stability challenges. Further comprehensive evaluations by Bjegovic et al. [42] expanded the understanding of these chemical interactions under severe freezing and deicing conditions.

A critical milestone in air-void stability research was achieved through the multi-part seminal work by Pigeon, Saucier, and colleagues. Pigeon et al. [43] established how key mixture parameters and supplementary cementitious materials, such as silica fume, govern early air-void characteristics (Part I). This was subsequently validated through field performance tests of superplasticized concrete by Saucier et al. [44] (Part III), and later integrated into a holistic structural analysis introducing a performance index and temperature-dependent stability variables by Saucier and Cameron [45] (Part V).

With the SCC, rheological behavior and air stability required independent verification due to the highly fluid nature of the matrix. Beaupré et al. [46] connected the specific rheology of SCC directly to its scaling resistance. Khayat [47] deeply explored the overall performance of air-entrained SCC, which laid the groundwork for identifying the precise mechanisms behind air-void stability and potential air loss during handling and casting in fluid systems [48].

Simultaneously, the widespread incorporation of supplementary materials altered the microstructural evolution of the paste matrix. Kjellsen and Atlassi [49] provided detailed insights into the complex pore structure of silica fume systems. This microstructural transformation was further quantified by Papadakis et al. [50], who modelled the hydration and carbonation kinetics of pozzolanic cements. Papadakis subsequently extended these predictive models to define the chemical activity of fly ash [51] and developed fundamental silica fume activity modelling [52] within modern cement systems. Ultimately, these cumulative microstructural and macrostructural insights formed the basis for comprehensive concrete durability design guidelines, as synthesized in the landmark work of Fagerlund [53]. Applying these criteria specifically to high-performance fluid mixtures, Persson [54,55] completed extensive long-term experimental programs confirming the overall frost resistance and multi-faceted durability of self-compacting concrete configurations.

From among the previously presented concepts involving the description of the relationship between the structure of the mix and its properties, the most interesting is the idea of Synthetic Dispersion Indicators (SDI). Dispersion indicators offer a comprehensive description, ranging from a sub-micro scale to a macro scale, of the relationship of the binder–aggregate relationship [56]. They were used to derive theoretical relations for the mix and hardened concrete features. Thus, they can specify two stages in the development process: shaping the properties of the matrix and shaping the properties of particles, and then the proportions of their mutual mixing.

The composite formula has many advantages:

- It identifies the mixture not from a technological point of view as a specific mixture of a specific amount of a particular type of ingredients, but from a physicochemical point of view, as a generalised system with a determined phase composition.
- It resigns from the technical description of the composition of the mixture (using quantitative parameters and qualitative ones, often in one form) in favour of a synthetic, numerical and, consequently, accurate description of its structure.

The features mentioned above account for the appropriate predispositions of such a description for all kinds of mathematical modelling. The only obstacle in their practical use may be the availability of parameters and characteristics indispensable to determine them. Suppose we adopt a concept of the development of self-compacting concrete based on the composite formula of concrete.

In that case, we can limit a large set of factors determining the process of venting the mix to three groups:

- Matrix, which the properties of cement paste will characterise;
- Particles, i.e., aggregate with its properties;
- Filling up the aggregate particles with a matrix—the proportions of the volume share of aggregate and cement paste in the mix.

The SDI concept consistently and logically connects elements from the micro-scale (cement paste) to the macro scale (mix). In addition, there are potential possibilities to expand this set with the elements of the sub-micro scale. For the above reasons, the proposed description of the concrete mix using SDI seems appropriate to examine the impact of self-compacting on the stability of its air entrainment.

In line with the theoretical assumptions of the idea of Synthetic Dispersion Indicators SDI and the composite formula of concrete mix, the system was divided into two levels:

- Dispersion system of cement paste;
- Dispersion system of aggregate.

The filling degree of aggregate with cement paste can be represented in the form of the indicator φ the ratio of the apparent volume of cement paste to the aggregate volume. As to the characteristics of the mix affecting the stability of its air entrainment, we must first consider the size of inter-grain spaces filled with cement paste. The size of these spaces is well described by the φ index of aggregate filling with cement paste. The said quantity comprises the effect of the ratio of mortar amount to aggregate volume and is presented by the following relation:

*φ* = *D*_m_/*D*_s_, (1)

*D*_m_—dispersion indicator of concrete mixture,
*D*_s_—dispersion indicator of crumb pile,
or

*φ* = V_z_/(*K* × *P*_dwk_), (2)

*K*—aggregate mass, kg,
*V*_z_—a volume of cement paste, m^3^,
*P*_dwk_—porosity of loosely stacked crumb pile, m^3^/kg.

The dispersion indicator of concrete mix *D*_m_, present in the expression (1), is given by the following expression [18]:

(3) $$D_{m}=\frac{V_{z}}{S_{wsk}K},$$

Here, *K* represents aggregate mass, kg, and

(4) $$V_{z}=\frac{C}{\rho_{c}}+\frac{M_{p}}{\rho_{p}}+W,$$

stands for absolute volume of cement paste.

The structure and properties of aggregate can be characterised by the dispersion index of crumb pile *D*_s_, which considers the aggregate, grain size, shape, type and size of the developed grain surface. It also includes the volume share of the continuous (air) phase to the aggregate’s inter-phase surface (developed surface).

The dispersion indicator of crumb pile *D*_s_ is expressed by the following relation:

(5) $$D_{s}=\frac{P_{dwk}}{S_{wsk}},$$

or

(6) $$D_{s}=\frac{\rho -\rho_{n}}{\rho \cdot \rho_{n}\cdot S_{wsk}},$$

where *P*_dwk_ is the specific porosity available for the water of a loosely stacked pile of the bulk density of *ρ*_nl_ and verified density of *ρ*_s_

(7) $$P_{dwk}=\frac{1}{\rho_{nl}}\left(1-\frac{\rho_{nl}}{\rho_{s}}\right)=\frac{1}{\rho_{nl}}-\frac{1}{\rho_{s}},\;m^{3}/kg,$$

(8) $$S_{wsk}=0.01\sum_{i}U_{i}\frac{K}{\overset{-}{d_{i}}\rho_{is}},\;m^{2}/kg,$$

where

*ρ*_nl_—bulk density, kg/m^3^,
*ρ*_s_—verified density, kg/m^3^,
*ρ*_is_—verified density of the fraction, kg/m^3^,
*U*_i_—weight share of the fraction in the pile, %,
*K*—grain shape factor,
$\overset{-}{d_{i}}$—average size of the fraction, kg.

Analogous to the crumb pile dispersion index is the cement paste dispersion index *D*_z_ representing the average thickness of water lagging of cement and dust grains in the cement paste.

Another important factor in terms of the air-entrainment stability of SCC involves the properties of cement paste. The said properties are expressed by the *D*_z_ index, which is determined by the following relationship:

(9) $$D_{z}=\frac{W}{C\cdot S_{wsc}+M_{p}S_{wsp}},\;m,$$

where

*W*—water volume in the cement paste, m^3^,
*S*_wsc_—specific surface of cement, m^2^/kg,
*S*_wsp_—specific surface of dust, m^2^/kg,
*C*—cement mass in the cement paste, kg,
*M*_p_—dust mass in the cement paste, kg.

For a given cement and dust, the *D*_z_ index can be presented in a more straightforward form generally known in concrete technology:

(10) $$D_{z}=\frac{W}{C+M_{p}},\;m,$$

which, although dimensionless, reflects the physical nature of the index. However, in the case of a self-compacting mix, the properties of cement paste are also affected by chemical additives, mineral additives and the type of cement. Therefore, the dispersion index of the cement paste does not entirely reflect the impact of cement paste properties on the self-compacting level of the self-compacting mixture, so appropriate modifications should be made in this respect.

The self-compacting process of concrete mixes, also affecting the stability of air-entrainment of the mix, is not yet entirely known. Ensuring the air-entrainment stability of the self-compacting concrete mix raises several unresolved questions. Therefore, the impact of self-compacting of the mix on air-entrainment stability should be verified experimentally, which is this article’s purpose.

#### 2.2.2. Research Methodology for Assessing the Influence of Synthetic Dispersion Indicators on SCC Air-Void Stability

The significance of the examined factors was evaluated with respect to individual response variables rather than a single generalised performance parameter. The analysed responses included air content after 5 min, air content after 15 min, absolute air loss between 5 and 15 min, fresh mixture density, hardened air content after 28 days, spacing factor L, micropore content A_300_ and specific surface α. This response-variable-specific approach was adopted to avoid overgeneralised statistical conclusions and to maintain a direct link between the experimental design and the measured properties.

The experimental programme was based on a five-level Graeco-Latin square design involving four principal factors: AEA dosage, binder type, water-to-binder ratio, and the volumetric paste-to-aggregate filling parameter *φ*. The qualitative relationship investigated experimentally was defined as

*P* = *F*(%*AEA*, *binder type*, *w*/*b*, *φ*) (11)

where *P* denotes the analysed response variable, %*AEA* is the dosage of air-entraining admixture expressed as a percentage of cement mass, binder type represents the cementitious system, *w*/*b* is the water-to-binder ratio, and *φ* is the volumetric filling ratio of aggregate with cement paste.

The factor levels used in the experimental design were as follows: %AEA = x1: 0.00, 0.05, 0.10, 0.15 and 0.20% by mass of cement; w/b = x2: 0.29, 0.32, 0.35, 0.38 and 0.41; binder type = x3: CEM I 32.5 R + 10% SF, CEM II/B-V 32.5 R, CEM II/B-S 32.5 R, CEM II/B-M 32.5 R and CEM III/A 32.5 N; and *φ* = x4: 1.1, 1.2, 1.3, 1.4 and 1.5.

The aggregate skeleton remained unchanged throughout the study and was represented by a constant aggregate dispersion index *D*_s_. Consequently, the design enabled the influence of paste composition, entrained air content, and aggregate filling conditions to be evaluated without modifying aggregate grading or packing characteristics. The resulting 25 mixtures generated by the Graeco-Latin square design are presented in Table 2 and Table 3.

The aggregates used to make the mixtures were made from washed river gravel of grain size up to 8 mm. The SCC mixtures intended for the main study were prepared with constant aggregate volume and grading, which means that the aggregate skeleton descriptor *D*_s_ was kept constant. Based on preliminary tests, the sand point was selected to obtain high deformability and a short flow time for the adopted mixture proportions. The most favourable sand point for the determined proportions was 0.49. The crumb pile was characterised by the dispersion index *D*_s_ = 0.00041 m. The air-entraining admixture was dosed according to the levels given in Table 2, while the superplasticizer dosage was adjusted to approach the target SCC slump-flow class.

### 2.3. Verifying the Impact of SDI on SCC Porosity

The assessment of the impact significance of the examined factors is closely related to statistical correlation. When applied in experimental research, the following can be distinguished: assessment of impact significance of the examined factors (significance assessment), which is conventionally defined by the concepts of qualitative correlation, linear correlation, and nonlinear correlation. The analysis of variance is the primary method of statistical assessment of the impact significance of the examined factors.

The assessment can be conventionally referred to as a qualitative correlation, as it allows us to determine whether a change in the values of the examined factors

- has a significant impact, or
- has no significant effect on the resultant factor.

And the function relationship is not specified here between the examined factors and the resulting factor (it is not known whether it is a linear or curvilinear correlation).

The basic concept of the analysis of variance is the variance defined as follows:

(12) $$\sigma^{2}(z)=\frac{\sum_{k=1}^{n}(z_{k}-\mu {)}^{2}}{n},$$

where:

*z*—resultant factor *z*,
*k*—ordinal variable,
*n*—number of measurements,
*μ*—population mean, μ = ${\overset{-}{z}}_{0}$,
${\overset{-}{z}}_{0}$—mean of the general population.

The variance $\sigma^{2}(z)$ can be the so-called corrected variance:

(13) $$S^{2}(z)=\frac{\sum_{k=1}^{n}(z_{k}-\overset{-}{z}{)}^{2}}{n-1},$$

where

$\overset{-}{z}$—mean of parallel measurements of the cardinality n = r,

(14) $$\overset{-}{z}=\frac{1}{n}\sum_{k=1}^{n}z_{k},$$

The expression in the denominator stands, in general, for the degrees of freedom f, whereas the numerator expression is the so-called sum of squared deviations from the average:

(15) $$SQ=\sum_{k=1}^{n}(z-\overset{-}{z})=\sum z^{2}-\frac{{\left(\sum z\right)}^{2}}{n}$$

We verify the adequacy of the linear model using the F-Snedecor test, checking the inequality:

(16) $$F_{R}=\frac{\overset{-}{Q_{R}}}{\overset{-}{Q_{WU}}}<F_{\alpha ,f_{2},f_{1}},$$

where

$\overset{-}{Q_{R}}$—“residual” mean square,

(17) $${\overset{-}{Q}}_{R}=\frac{SQ_{R}}{f_{R}},$$

$\overset{-}{Q_{WU}}$—mean square “inside the systems”,

(18) $${\overset{-}{Q}}_{WU}=\frac{SQ_{WU}}{{f_{W}}_{U}},$$

where:

SQ—sum of squared deviations from the average,
*F_.R._*—the calculated quotient of “residual variance”,
$F_{\alpha ,f_{2},f_{1}}$—tabular quotient of variance, *f*_2_—degrees of freedom of the numerator of the quotient (*f_2_* = *f_R_*),
*f*_2_—degrees of freedom of the denominator of the quotient (*f*_2_ = *f_R_*),
α—significance level.

If the above inequality is satisfied, interaction is negligible, i.e., the adopted linear model for the research programme is correct. Failure to meet the above inequality means that the model is inadequate, i.e., the interactions of the factors are significant, and further conclusions about the significance of the factors is questionable. The executors of the experimental research often forget the above condition. Neglecting the impact of interaction, i.e., elimination of the nonlinear model in favour of the linear model, is a significant simplification that should be fully justified in the substantive analysis of the research object. Table 4 summarises the results of the three-fold measurement of *g* for each of the series of mixtures. After the appropriate calculations, we obtain

$${\mathrm{SQ}}_{R}=0.32\;{\overset{-}{Q}}_{R}=\frac{SQ_{R}}{f_{R}}=0.08\;{\overset{-}{Q}}_{WU}=\frac{SQ_{WU}}{{f_{W}}_{U}}=0.36$$

$$F_{R}=\frac{{\overset{-}{Q}}_{R}}{\overset{-}{Q_{WU}}}=0.22<F_{0.05-4-8}=3.84<F_{0.01-4-8}=7.01$$

The impact of the interaction is negligible at the significance level of α ≤ 0,05, and a linear model can be adopted.

As shown in Table 4, the three-fold measurement of the workability-related parameter *g* indicates that the response of the self-compacting concrete mixes depended on both the dosage level *x*_1_ and the cement type *x*_2_. The highest cumulative value of *k* was obtained for the system with CEM I 32.5 R + 10% SF (*k* = 7.12), whereas the lowest value was recorded for CEM III 32.5 (*k* = 3.05). In terms of the *x*_1_ factor, the highest total values were observed for the levels 0.10 and 0.15, which suggests that the workability-related response was most pronounced in this intermediate range of the analyzed variable.

### 2.4. Assessment of the Impact of Examined Factors on Test Results

Since the linear model was adopted, the impact significance of the factors under consideration can be assessed using the F test:

(19) $$F=\frac{S^{2}(z_{2})}{S^{2}(z_{1})},$$

where:

*S*^2^(*z*_1_)—estimated variance for one selected state of the object,
*S*^2^(*z*_2_)—estimated variance for the second selected state of the object,

The further course of calculations is limited to comparing the quotient F (25) calculated value with the tabular value $F_{\alpha ,f_{1},f_{2}}$. If *F* > $F_{\alpha ,f_{1},f_{2}}$, then it gives a significant result. In other words, the given impact of factor x_i_ is significant and should be included in the future mathematical model. However, if F < $F_{\alpha ,f_{1},f_{2}}$, then at the assumed significance level α the test gives an insignificant result, and the tested factor x_i_ has no effect on the resulting factor z; it can be eliminated from further testing.

## 3. Research Results and Discussion

### 3.1. General Results and Their Analysis

Table 5 and Table 6 summarise the fresh-state results for the 25 SCC mixtures, including slump-flow time, slump-flow diameter, air content after 5 and 15 min, air loss and density. Table 7 presents the hardened air-void parameters determined after 28 days. These three tables form the primary experimental basis for interpreting the transition from fresh-air entrainment to hardened pore structure.

The measured slump-flow values are reported in Table 5 and ranged from 50 to 79 cm. Therefore, slump flow was not treated as a constant rheological parameter in the interpretation but as an experimentally observed workability response reflecting differences in mixture behaviour. The workability results in Table 5 show that the mixtures remained within a broad SCC-type flow range, but the target slump-flow value of approximately 675 mm was not achieved identically in all compositions. The measured slump-flow diameter varied from 50 to 79 cm and the T_50_ time from 2 to 13 s. This variability is important because it indicates different empirical workability states and potentially different bubble mobility conditions; however, without direct rheometry these observations must not be converted into quantitative yield stress or plastic-viscosity values.

The measured slump-flow values are reported in Table 5 and are discussed as experimental responses rather than as identical constant values. The lowest slump-flow diameters were observed for mixtures 4, 21, 24 and 25, whereas the largest values were obtained for mixtures 6, 11 and 19. These differences show that the combined effect of binder type, w/b, AEA dosage and φ influenced the fresh-state behaviour. Since direct rheometry was not performed, the discussion does not assign quantitative yield stress or plastic viscosity values to these mixtures.

The results presented in Table 6 show that total air content in fresh mixtures varied from approximately 1.6% to 8.6%, depending on composition. The comparison of air content measured after 5 and 15 min provides an early laboratory measure of air retention. The observed loss ranged from 0.41 to 1.12 percentage points, indicating that all mixtures experienced some reduction in measured air content during the first 15 min. This interval is informative for comparing mixtures under controlled laboratory conditions, but it should not be interpreted as a full simulation of transport, pumping, casting or setting.

The air-content results in Table 6 show that fresh air content after 5 min ranged from 1.6% to 8.6%. The difference between air content after 5 and 15 min ranged from 0.41 to 1.12 percentage points. This confirms that air loss occurred in every mixture, although the magnitude of loss was moderate under the laboratory time interval used. The 5–15 min comparison should therefore be interpreted as early air retention, not as complete technological stability during transport or setting.

The density values in Table 6 are consistent with the air-content measurements: mixtures with high air contents generally showed lower density. Examples include mixtures 16 and 21, which had high air contents and relatively low densities. These observations confirm the internal consistency of the fresh-state measurements and justify their use in further interpretation of hardened pore structure.

The pore structure parameters presented in Table 7 provide further insight into the relationship between fresh mix behaviour and hardened concrete microstructure. The total air content in hardened concrete ranges from approximately 1.2% to 8.6%, which corresponds well with the values measured in fresh mixtures. This confirms that, despite some air loss, the air-void system is largely preserved during the transition from fresh to hardened state.

The hardened air-void parameters in Table 7 demonstrate that total hardened air content ranged from 1.2% to 8.59%, while the spacing factor *L* varied from 0.13 to 0.44 mm. The lowest spacing factors were obtained for mixtures 21 and 16, whereas mixtures 9, 11 and 2 had the highest *L* values. This confirms that the quality of the air-void system cannot be evaluated solely from total air content.

Specific surface α and micropore content *A*_300_ also varied substantially between mixtures. For example, mixture 21 combined high hardened air content with the lowest spacing factor and the highest specific surface, whereas mixture 9 had low hardened air content and the highest spacing factor. These contrasts support the need to analyse *A*, *L*, *A*_300_ and *α* together rather than relying on one isolated descriptor.

**Figure 1:**
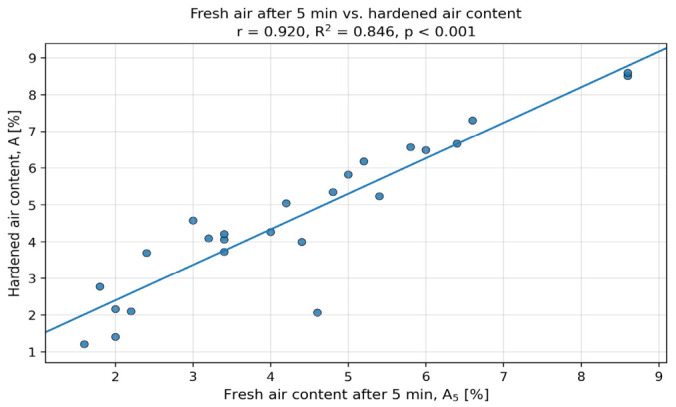
Relationship between air content in fresh concrete after 5 min and total air content in hardened concrete.

**Figure 2:**
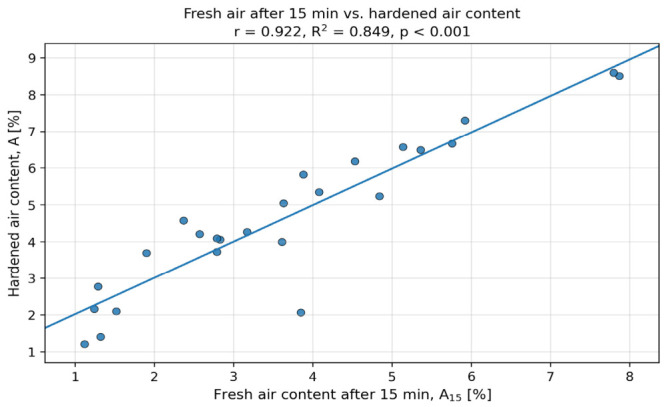
Relationship between air content in fresh concrete after 15 min and total air content in hardened concrete.

The relationships between fresh air content and hardened air content are shown in Figure 1 and Figure 2. The correlation coefficients reported in the figures indicate strong correspondence between air content after mixing, air content after 15 min and hardened air content after 28 days. This supports the conclusion that early retained air is a useful indicator of the final air-void system within the investigated mixtures.

**Figure 3:**
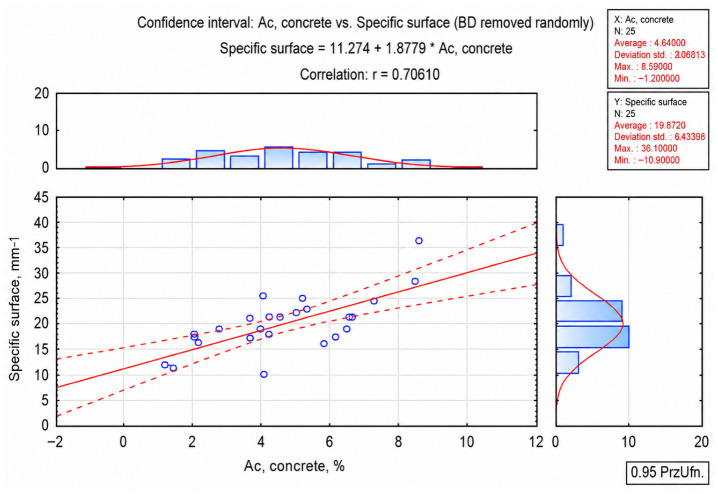
Relationship between air content in fresh concrete (immediately after mixing) and specific surface of pores in the hardened concrete.

**Figure 4:**
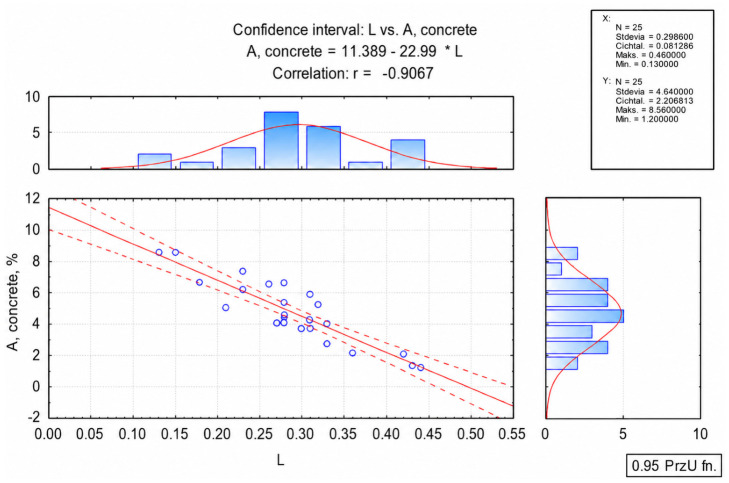
Relationship between pore spacing index *L* and air content *A* in hardened concrete.

**Figure 5:**
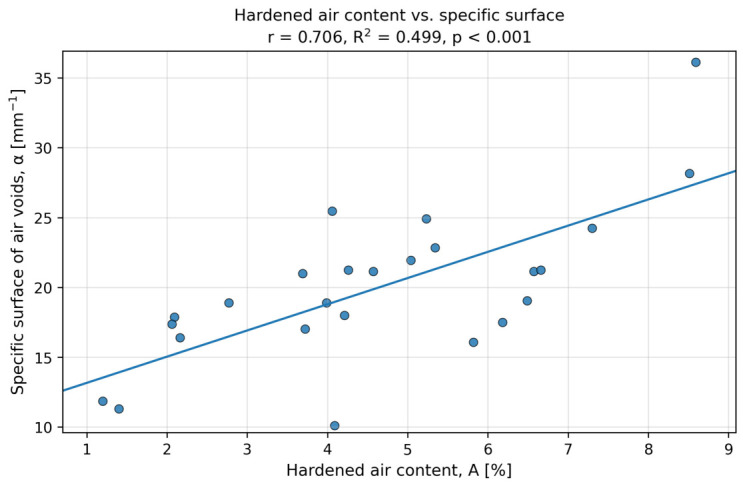
Relationship between air content *A* and specific surface *α* of pores in hardened concrete.

**Figure 6:**
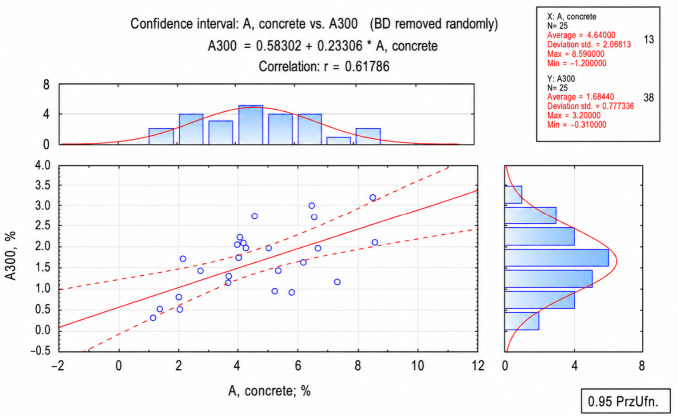
Relationship between the total air content and the content of pores of the diameter lower than 0.300 mm in hardened concrete.

Figure 3, Figure 4, Figure 5 and Figure 6 present relationships between fresh air content, hardened air content, specific surface, spacing factor and micropore content. These figures show that the air-void system is internally linked but not governed by total air content alone. Figure 4 illustrates that lower spacing factors are associated with more favourable pore distribution, while Figure 5 and Figure 6 show that specific surface and micropore content respond nonlinearly to total air content.

To strengthen the interpretation of Table 6 and Table 7, additional derived indicators were calculated and are presented in Table 8. These indicators do not introduce new experimental data. They are calculated directly from the measured values and help clarify air retention and transfer from the fresh to hardened state.

The derived indicators include the relative air-retention ratio after 15 min (R_15_), relative air loss, the ratio between hardened air content and air content after 15 min (*A*/*A*_15_), and the micropore fraction in total hardened air content (*A*_300_/*A*). These values directly address the question of whether the entrained air was merely present in the fresh mixture or effectively retained as a measurable hardened air-void system.

Figure 1 and Figure 2 confirm the strong fresh-to-hardened relationship, while Table 8 provides mixture-by-mixture evidence of the retention and transfer ratios. In most mixtures, R_15_ exceeded 0.75, showing that most of the initially entrained air remained in the fresh mixture after 15 min. However, the *A*/*A*_15_ ratio varied, confirming that the fresh-to-hardened transition was not identical for all cementitious systems.

Figure 3, Figure 4, Figure 5 and Figure 6 compare hardened pore-structure descriptors. The observed relationships are consistent with the numerical data in Table 7 and Table 8. They demonstrate that the development of a refined pore system depends on the simultaneous presence of retained air and favourable pore distribution. This is why the interpretation focuses on the combined assessment of *A*, *L*, *A*_300_ and *α*.

The main quantitative relationships were evaluated by Pearson correlation. Fresh air content after 5 min and after 15 min correlated strongly with hardened air content (*A*_5_ vs. *A*: r = 0.920, R^2^ = 0.846, *p* < 0.001; *A*_15_ vs. *A*: r = 0.922, R^2^ = 0.849, *p* < 0.001). Hardened air content was strongly and inversely correlated with spacing factor (*A* vs. *L*: r = −0.907, R^2^ = 0.822, *p* < 0.001), confirming that increased retained air was generally associated with shorter pore spacing. Moderate-to-strong correlations were also found for A vs. *A_300_* (r = 0.618, *p* = 0.001) and *L* vs. α (r = −0.683, *p* < 0.001).

The statistical evaluation was carried out using ANOVA based on the Graeco-Latin square experimental design. The purpose of this analysis was not to prove a universal predictive model but to identify which main factors were statistically relevant for each response variable within the investigated material range.

Before interpreting the main effects, the adequacy of the adopted linear model was checked according to the procedure described in Section 2.3. This test supports the use of the Graeco-Latin square design as a screening approach. Neverheless, the possible existence of physicochemical interactions is not excluded; it is discussed separately as a mechanistic interpretation.

**Figure 7:**
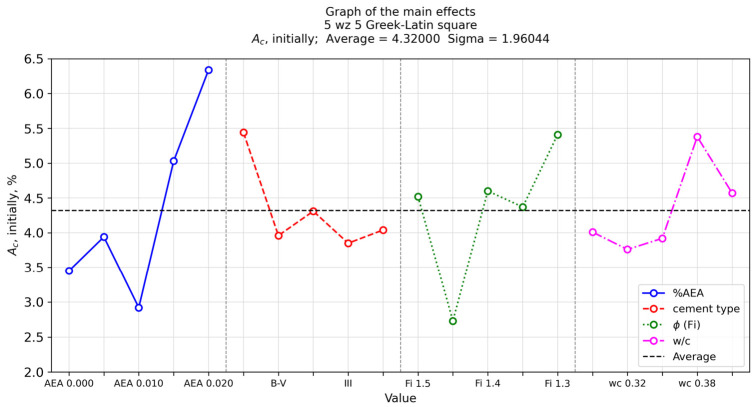
Graph of the main effects of % AEA, type of cement, *φ* (FI), w/c on the initial air-entrainment of the self-compacting concrete mix.

**Figure 8:**
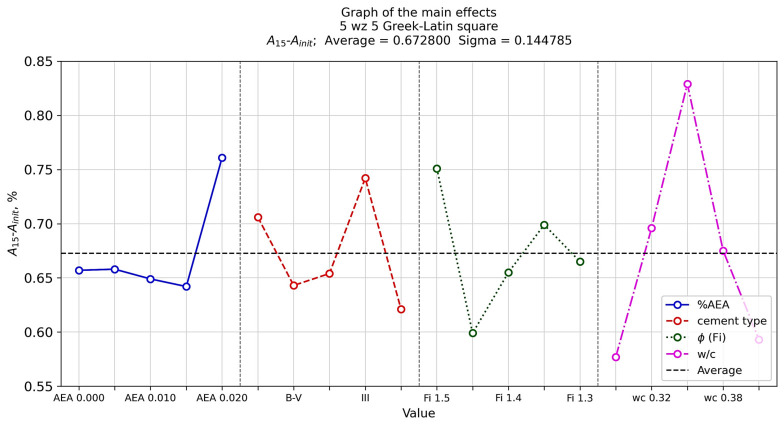
Graph of the main effects of % AEA, type of cement, *φ* (Fi), w/c on the change in the air-entrainment of the self-compacting concrete mix.

**Figure 9:**
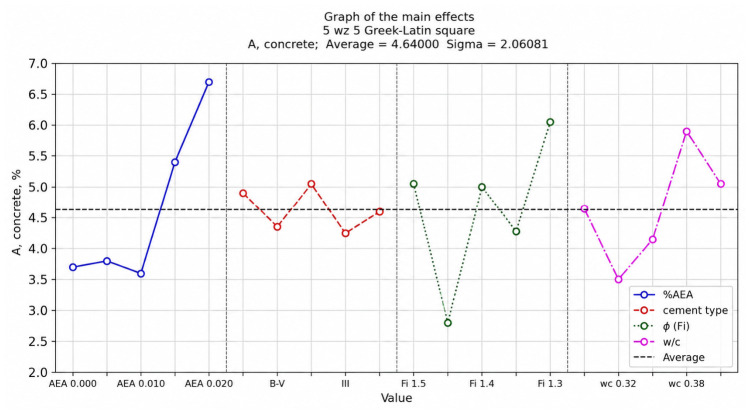
Graph of the main effects of % AEA, type of cement, *φ* (Fi), w/c on the total air content in the hardened self-compacting concrete.

**Figure 10:**
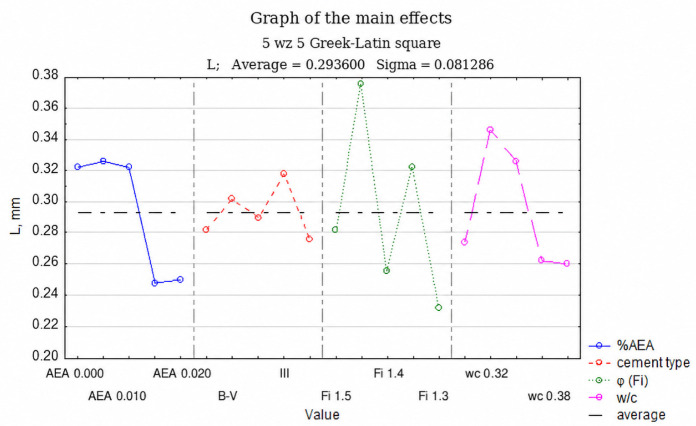
Graph of the main effects of % AEA, type of cement, *φ* (Fi), w/c on the pore spacing indicator in the hardened self-compacting concrete.

**Figure 11:**
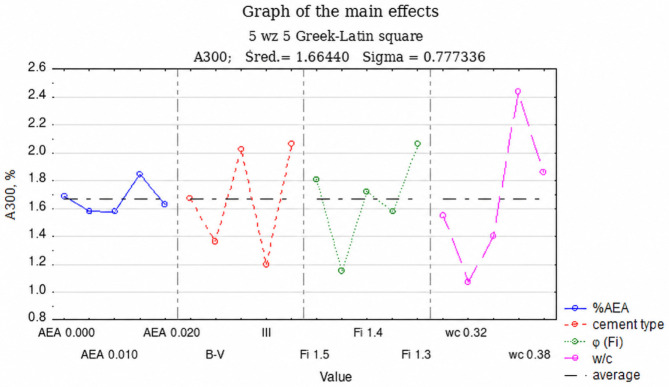
Graph of the main effects of % AEA, type of cement, *φ* (Fi), w/c on the content of A_0,300_ pores in the hardened self-compacting concrete.

**Figure 12:**
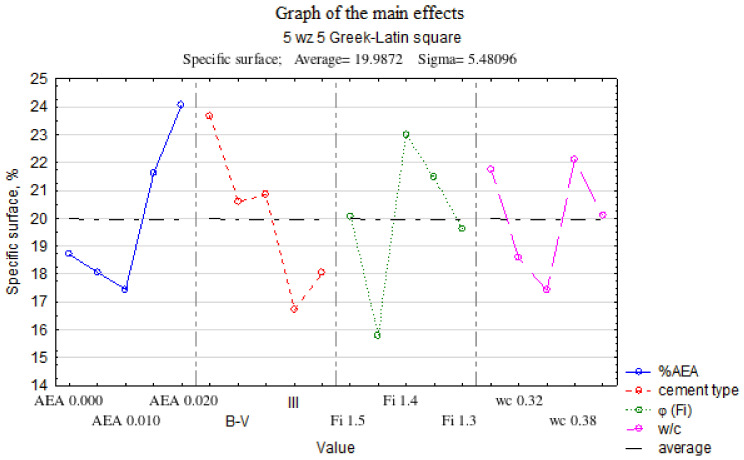
Graph of the main effects of % AEA, type of cement, *φ* (Fi), w/c on the specific surface of pores in the hardened self-compacting concrete.

The results in Table 9, Table 10, Table 11, Table 12, Table 13 and Table 14 and Figure 7, Figure 8, Figure 9, Figure 10, Figure 11 and Figure 12 show that the statistical significance of the factors depends strongly on the analysed response variable. Therefore, the revised interpretation avoids the statement that one or two factors are universally dominant for the whole air-void system.

For the initial fresh air content *A*_5_ (Table 9), none of the analysed factors reached the conventional significance threshold of *p* < 0.05. Binder type showed a near-threshold tendency (F = 3.122, *p* = 0.080, partial η^2^ = 0.609), whereas AEA dosage, w/b and *φ* were not statistically significant. This result indicates that the measured fresh air content was not governed by AEA dosage alone, but by the response of the complete SCC system.

For early air loss between 5 and 15 min (Table 10), w/b reached statistical significance (F = 4.190, *p* = 0.040, partial η^2^ = 0.677). This finding should be interpreted cautiously, because the residual normality test indicated a non-normal residual distribution for this response. Nevertheless, the result supports the physically reasonable interpretation that water-to-binder ratio and the related paste state may influence early air escape under laboratory conditions.

For hardened air content A (Table 11), no main factor reached *p* < 0.05. Binder type showed the strongest near-threshold tendency (F = 3.345, *p* = 0.069, partial η^2^ = 0.626), followed by w/b (F = 2.708, *p* = 0.107, partial η^2^ = 0.575). Consequently, hardened air content should not be attributed solely to AEA dosage in this dataset. The very strong correlation between fresh and hardened air content indicates that early retained air is the best direct descriptor of final air content.

For the spacing factor *L* (Table 12), no factor reached statistical significance at α = 0.05. The strongest trend was associated with w/b (F = 2.508, *p* = 0.125, partial η^2^ = 0.556), whereas φ did not reach significance in the recalculated ANOVA. This result is important because it prevents overstatement of the SDI interpretation: aggregate-paste geometry remains mechanistically relevant, but the present dataset supports it as a physically plausible explanatory factor rather than as a statistically confirmed main effect for *L*.

For micropore content *A*_300_ (Table 13), w/b was statistically significant (F = 4.058, *p* = 0.044, partial η^2^ = 0.670). For specific surface α (Table 14), w/b showed a near-threshold tendency (F = 3.540, *p* = 0.060, partial η^2^ = 0.639). These results indicate that pore refinement is more closely associated with paste-related conditions than with total air content alone.

The overall statistical interpretation is summarised in Table 15. The table separates statistically significant effects, near-threshold tendencies and non-significant effects, directly addressing the need for a more nuanced interpretation of the ANOVA data.

Consequently, the results support a bounded conclusion: the transition from fresh air to hardened pore structure is strongly correlated, but the individual design factors do not act as universal predictors of all air-void descriptors. The SDI approach is best understood as a structured explanatory framework linking composition, early retention and hardened pore geometry. It should not be presented as a validated model for external prediction beyond the investigated material range.

Overall, the recalculated ANOVA, the correlations and the experimental data presented in Table 5, Table 6 and Table 7 indicate that air-void behaviour in SCC is governed by coupled effects. Fresh-air retention is strongly related to hardened air content, while pore spacing and pore refinement require separate descriptors. The present results do not justify a simplified statement that AEA dosage or φ alone controls the whole air-void system.

From a scientific perspective, these findings strengthen the manuscript because they show that the SCC air-void system demonstrate a coupled dispersion problem rather than a simple dosage-response problem. The same total air content may correspond to different spatial pore distributions; therefore, *A*, *L*, *A*_300_ and α must be interpreted jointly. This conclusion directly addresses the reviewers’ concern that mechanistic discussion should not imply unverified causality.

The results presented in Table 5, Table 6, Table 7 and Table 8 and Figure 1, Figure 2, Figure 3, Figure 4, Figure 5 and Figure 6 show that air-void stability in SCC is governed by the combined effect of early air retention and hardened pore distribution. The data do not support a simplified interpretation based only on AEA dosage or total air content. Instead, the final pore system must be evaluated using air content, spacing factor, micropore content and specific surface together. This interpretation is further supported by the statistical synthesis of the ANOVA results presented in Table 16.

The statistical results presented in Table 9, Table 10, Table 11, Table 12, Table 13 and Table 14 show that the main effects depend on the selected response variable. In addition to the F-values and *p*-values, partial η2 values were reported to describe the design-specific effect size. Within the recalculated dataset, w/b was significant for early air loss Δ*A* (partial η^2^ = 0.677) and micropore content *A*_300_ (partial η^2^ = 0.670), while binder type showed near-threshold tendencies for A5 and A. These effects should be interpreted as screening results, not as universal material constants.

The variability of slump-flow diameter and flow time in Table 5 shows that the mixtures did not behave as rheological identical systems. Since no direct rheometric measurements were performed, this study cannot quantify yield stress, plastic viscosity, thixotropy or structural build-up. The discussion therefore uses slump flow only as a workability indicator and identifies direct rheology as a necessary next stage of research.

Although φ was not statistically significant for *L* in the recalculated ANOVA, the role of aggregate-paste geometry remains physically relevant. The volumetric filling of aggregate voids with cement paste modifies interparticle spacing and may alter bubble migration paths, coalescence probability and local pore distribution. Therefore, mixtures with comparable total air contents may still develop different spacing factors. This interpretation should be treated as a mechanistic hypothesis supported by the SDI framework and by the observed pore-structure contrasts, not as a statistically proven universal law.

The pore-structure data in Table 7 confirm that mixtures with similar total air contents may have different spacing factors and specific surfaces. For example, high air content accompanied by a low spacing factor indicates a more refined and spatially effective air-void system, whereas low air content combined with a high spacing factor indicates an unfavourable distribution. Table 8 further shows that air retained in the fresh mixture was not always transferred into the hardened pore system in the same proportion. This is why total air content alone cannot serve as a sufficient descriptor of the air-void system.

Binder type and water-to-binder ratio should not be described as having no influence in a general sense. Within the adopted Graeco-Latin square design, their statistical influence depended on the selected response variable. Their indirect effects through particle-size distribution, surface chemistry, paste water availability, hydration kinetics and admixture compatibility remain physically plausible. This distinction between statistical confirmation and mechanistic plausibility was introduced to avoid causal overinterpretation.

The correlations calculated from Table 6 and Table 7 confirm that early fresh-air measurements are useful indicators of hardened air content. The correlation between air content after 5 min and hardened air content was strong and statistically significant (r = 0.920, R^2^ = 0.846, *p* < 0.001), and the corresponding relationship for air content after 15 min was similarly strong (r = 0.922, R^2^ = 0.849, *p* < 0.001). Hardened air content was also strongly and inversely correlated with spacing factor L (r = −0.907, R^2^ = 0.822, *p* < 0.001), confirming that higher total air content generally contributed to a denser air-void distribution within the investigated range. However, the 5–15 min interval used in this study is limited and should be interpreted as an early-stage laboratory air-retention test rather than a representation of transport, pumping, casting or setting under construction conditions.

The results also have implications for durability-oriented interpretation, but only within clear limits. Parameters such as spacing factor, micropore content and specific surface are commonly used to assess the potential quality of an air-void system. Nevertheless, no freeze–thaw testing was performed in this study. Therefore, this manuscript does not claim confirmed freeze–thaw resistance; it only identifies pore-structure characteristics that may be relevant for future durability verification.

The scientific contribution of this work is the integrated interpretation of early air retention, hardened air-void structure and dispersion-geometry descriptors under a statistically structured experimental programme. The novelty is not the proposal of a universal model, but the demonstration that fresh-air retention, *A*, *L*, *A*_300_ and α must be interpreted as a coupled set of descriptors when assessing air-void stability in SCC.

The strong correlations between air content in fresh mixtures and hardened concrete (Figure 1 and Figure 2) confirm that the air-void system is largely conditioned at the early stage after mixing. At the same time, the observed 5–15 min air loss indicates that dynamic processes such as bubble migration, coalescence and escape continue to occur before setting. This dual observation is central to this manuscript: fresh air content is a useful indicator, but it does not replace hardened pore-structure analysis.

The experimental results presented in Table 5, Table 6, Table 7 and Table 8, together with the ANOVA results in Table 9, Table 10, Table 11, Table 12, Table 13 and Table 14 and the synthesis in Table 15, indicate that air-void stability can be interpreted using a dispersion-geometry framework. However, this framework must be treated as semi-empirical and limited to the investigated materials and mixture range.

Based on these observations, the early change in air content may be expressed conceptually as a function of the factors controlling bubble formation, mobility and geometrical confinement:

Δ*A* = *f*(*AEA*, *φ*, *D*_z_, *D*_s_), (20)

where ΔA represents air loss over time, AEA is the dosage of air-entraining admixture, φ is the volumetric filling ratio of aggregate with cement paste, D_z_ is the dispersion-related descriptor of the paste phase, and D_s_ is the constant aggregate-skeleton descriptor used in the present experimental programme.

Because the ANOVA results do not confirm all factors as statistically significant for all response variables, this expression should not be interpreted as a validated predictive equation. It is a compact representation of the variables considered in the experimental design.

In a simplified interpretative form, the early air-loss tendency may be written as

Δ*A* = *f*(*AEA*, *φ*, *D_z_*, *D_s_*), (21)

where k1 is a proportionality coefficient, α and β are empirical sensitivity coefficients, and η represents the effective resistance of the paste phase to bubble motion. These parameters were not independently identified in this study; therefore, Equation (21) is used only to express the assumed direction of influence.

The hardened pore structure may be described conceptually by linking the spacing factor with total hardened air content and geometrical confinement:

*L *=* k*2⋅A − α⋅φ − γ (22)

where L is the spacing factor, A is the total hardened air content, k2 is a proportionality coefficient, α expresses the sensitivity of the spacing factor to the total hardened air content, and γ expresses the assumed sensitivity of the pore spacing to geometrical confinement. This relation is consistent with the strong inverse correlation between *A* and *L* and with the mechanistic role assigned to φ in the SDI framework. However, because *φ* was not statistically significant for *L* in the recalculated ANOVA, Equation (22) must be treated strictly as an interpretative relation requiring independent validation.

The proposed framework therefore has interpretative rather than predictive status. It helps organise the experimental evidence and explains why total air content alone is insufficient to describe the air-void system in SCC. Its coefficients were not calibrated independently, and the equations should not be used for mixture design outside the investigated range.

Further development of the model requires independent validation, direct rheological measurements, longer air-retention monitoring, bubble-size analysis in the fresh state, and direct durability tests, especially freeze–thaw resistance.

### 3.2. Limitations of the Study

This study has several limitations that were considered in the revised interpretation. First, slump-flow diameter and *T*_50_ time were used as empirical workability indicators, but direct rheological parameters were not measured. Second, the 5–15 min interval describes early laboratory air retention only and does not represent transport, pumping, casting or setting. Third, hardened air-void parameters were obtained from two determinations and should be treated as comparative descriptors rather than a full durability-classification dataset. Fourth, the Graeco-Latin square design is efficient for screening main effects, but it cannot fully identify all interactions between binder chemistry, PCE, AEA and paste volume. Fifth, no direct freeze–thaw tests were performed; therefore, the article discusses durability-related descriptors but does not claim verified freeze–thaw resistance. Consequently, the conceptual relation expressed in Equation (22) should be treated only as an interpretative framework and not as a predictive durability model.

## 4. Conclusions

The experimental programme demonstrated that air-void stability in SCC must be interpreted through the combined analysis of early fresh-air retention and hardened air-void structure. This study shows that total air content alone is not sufficient for evaluating the quality of the pore system because mixtures with comparable air contents may differ in spacing factor, micropore content and specific surface.

The AEA dosage range used in this study was 0.00–0.20% by mass of cement and was consistently applied in the Graeco-Latin square design. The measured slump-flow diameter ranged from 50 to 79 cm, confirming that workability varied between mixtures and should be treated as an experimental response rather than a fixed rheological condition.

Air loss between 5 and 15 min ranged from 0.41 to 1.12 percentage points. These values describe early laboratory air retention and should not be extrapolated directly to field transport, pumping, casting or setting. The derived indicators in Table 8 improve transparency by showing both relative retention and fresh-to-hardened air transfer.

The hardened air-void system could not be described by total air content alone. The spacing factor ranged from 0.13 to 0.44 mm, and A_300_ and α varied substantially. The strong inverse correlation between *A* and *L* indicates that pore distribution and pore refinement are essential descriptors of the hardened air-void system.

The ANOVA results require a response-variable-specific interpretation. In the recalculated dataset, w/b was statistically significant for early air loss Δ*A* (F = 4.190, *p* = 0.040; partial η^2^ = 0.677) and micropore content *A*_300_ (F = 4.058, *p* = 0.044; partial η^2^ = 0.670), whereas binder type showed near-threshold tendencies for fresh and hardened air contents. No individual factor should be presented as a universal predictor of all air-void descriptors.

AEA dosage and φ remain physically important variables in the experimental design, but their influence should be interpreted within the limits of the present dataset. AEA dosage contributes to air generation, while φ provides a geometrical descriptor of aggregate-paste filling; however, the statistical evidence does not justify treating either variable as a general standalone control parameter for the whole pore system.

The proposed dispersion-geometry model should be regarded as a semi-empirical explanatory framework limited to the investigated materials and mixture range. Its value lies in organising the relationships between mixture composition, early air retention and hardened pore geometry. It is not an externally validated predictive model and should not be used for mixture design without further calibration.

Direct durability claims were not made because freeze–thaw resistance was not tested. Future studies should combine the present approach with direct rheological measurements, longer-term air-retention monitoring, fresh-state bubble-size analysis, image-based pore-structure validation and freeze–thaw testing. Such work is necessary to convert the present descriptor-based interpretation into a durability-validated model.

## Figures and Tables

**Table 1:** Material characterisation used in the study.

Material	Type/Standard	Chemical Composition (wt.%)	Physical Properties
Cement CEM I	EN 197-1 [27]	CaO ~63, SiO_2_ ~20, Al_2_O_3_ ~5, Fe_2_O_3_ ~3	Blaine ~350–400 m^2^/kg, density ~3.15 g/cm^3^
Cement CEM II/B-V	EN 197-1	CaO ~55–60, SiO_2_ ~25–30	Blaine ~350 m^2^/kg
Cement CEM II/B-S	EN 197-1	CaO ~50–55, SiO_2_ ~30–35	Blaine ~360 m^2^/kg
Cement CEM II/B-M	EN 197-1	Variable (multi-component)	Blaine ~350 m^2^/kg
Cement CEM III/A	EN 197-1	CaO ~40–50, SiO_2_ ~35–40	Blaine ~400 m^2^/kg
Aggregate (0–8 mm)	Natural river aggregate	SiO_2_ dominant (>90%)	Density ~2.65 g/cm^3^, continuous grading
Water	EN 1008 [28]	—	Density 1.0 g/cm^3^
Superplasticizer (PCE)	Polycarboxylate ether	Organic polymer	Liquid, ~30–40% solids
Air-entraining admixture (AEA)	Surfactant-based	Organic compounds	Liquid

**Table 2:** Composition of self-compacting mixtures.

Series No.	*φ*	w/b	w/c	AEA % Mass of Cement [m.C]	Cement Type
1	1.2	0.32	0.46	0.00	CEM II/B-V
2	1.3	0.35	0.51	0.05	CEM II/B-V
3	1.4	0.38	0.55	0.10	CEM II/B-V
4	1.5	0.41	0.60	0.15	CEM II/B-V
5	1.1	0.29	0.42	0.20	CEM II/B-V
6	1.4	0.35	0.51	0.00	CEM II/B-M
7	1.5	0.38	0.55	0.05	CEM II/B-M
8	1.1	0.41	0.60	0.10	CEM II/B-M
9	1.2	0.29	0.42	0.15	CEM II/B-M
10	1.3	0.32	0.46	0.20	CEM II/B-M
11	1.5	0.29	0.42	0.00	CEM II/B-S
12	1.1	0.32	0.46	0.05	CEM II/B-S
13	1.2	0.35	0.51	0.10	CEM II/B-S
14	1.3	0.38	0.55	0.15	CEM II/B-S
15	1.4	0.41	0.60	0.20	CEM II/B-S
16	1.2	0.41	0.60	0.05	CEM III/A
17	1.1	0.38	0.55	0.00	CEM III/A
18	1.3	0.29	0.42	0.10	CEM III/A
19	1.4	0.32	0.46	0.15	CEM III/A
20	1.5	0.35	0.50	0.20	CEM III/A
21	1.3	0.41	0.60	0.00	CEM I + SF
22	1.4	0.29	0.42	0.05	CEM I + SF
23	1.5	0.32	0.46	0.10	CEM I + SF
24	1.1	0.35	0.51	0.15	CEM I + SF
25	1.2	0.38	0.55	0.20	CEM I + SF

**Table 3:** Variability of factors adapted to the requirements of the experiment planning methodology of ANOVA.

	k	CEM II/B-S 32.5 R	CEM II/B-V 32.5 R	CEM II/B-M 32.5 R	CEM III/A 32.5 N	CEM I 32.5 R + 10% SF
w						
0		D	A	C	E	B
		*φ* = 1.5 w/b = 0.29	*φ* = 1.2 w/b = 0.32	*φ* = 1.4 w/b = 0.35	*φ* = 1.1 w/b = 0.38	*φ* = 1.3 w/b = 0.41
0.05		E	B	D	A	C
		*φ* = 1.1 w/b = 0.32	*φ* = 1.3 w/b = 0.35	*φ* = 1.5 w/b = 0.38	*φ* = 1.2 w/b = 0.41	*φ* = 1.4 w/b = 0.29
0.10		A	C	E	B	D
		*φ* = 1.2 w/b = 0.35	*φ* = 1.4 w/b = 0.38	*φ* = 1.1 w/b = 0.41	*φ* = 1.3 w/b = 0.29	*φ* = 1.5 w/b = 0.32
0.15		B	D	A	C	E
		*φ* = 1.3 w/b = 0.38	*φ* = 1.5 w/b = 0.41	*φ* = 1.2 w/b = 0.29	*φ* = 1.4 w/b = 0.32	*φ* = 1.1 w/b = 0.35
0.20		C	E	B	D	A
		*φ* = 1.4 w/b = 0.41	*φ* = 1.1 w/b = 0.29	*φ* = 1.3 w/b = 0.32	*φ* = 1.5 w/b = 0.35	*φ* = 1.2 w/b = 0.38

**Table 4:** Total results of the three-fold measurement of the workability-related parameter *g* of self-compacting concrete mixes [Nm].

k		x_2_					w
	w	CEM II 32.5 R B-S	CEM II 32.5 R B-V	CEM II 32.5 R B-M	CEM III 32.5	CEM I 32.5 R + 10% SF	
x_1_	0	0.045 D	0.153 A	0.082 C	1.482 E	1.563 B	3.36
	0.05	0.180 E	0.633 B	1.182 D	1.203 A	1.290 C	4.49
	0.10	1.269 A	1.512 C	1.392 E	0.147 B	1.224 D	5.54
	0.15	1.401 B	1.563 D	0.960 A	0.075 C	1.533 E	5.53
	0.20	1.437 C	1.092 E	0.192 B	0.148 D	1.512 A	4.38
k		4.33	4.95	3.81	3.05	7.12	23.3

**Table 5:** Consistency and workability indicators of the self-compacting mixture.

Series No.	*φ*	w/b	AEA	Cement Type	Slump Flow Time Up to 50 cm	Slump Flow Diameter
	-	-	% Mass of Cement [m.C]	-	s	cm
1	1.2	0.32	0.00	CEM II/B-V	6	73
2	1.3	0.35	0.05	CEM II/B-V	5	72
3	1.4	0.38	0.10	CEM II/B-V	6	58
4	1.5	0.41	0.15	CEM II/B-V	5	50
5	1.1	0.29	0.20	CEM II/B-V	4	71
6	1.4	0.35	0.00	CEM II/B-M	5	77
7	1.5	0.38	0.05	CEM II/B-M	9	71
8	1.1	0.41	0.10	CEM II/B-M	6	67
9	1.2	0.29	0.15	CEM II/B-M	8	70
10	1.3	0.32	0.20	CEM II/B-M	8	73
11	1.5	0.29	0.00	CEM II/B-S	6.5	79
12	1.1	0.32	0.05	CEM II/B-S	7	73
13	1.2	0.35	0.10	CEM II/B-S	7	70
14	1.3	0.38	0.15	CEM II/B-S	5	66
15	1.4	0.41	0.20	CEM II/B-S	4.5	63
16	1.2	0.41	0.05	CEM III/A	6	63
17	1.1	0.38	0.00	CEM III/A	6	67
18	1.3	0.29	0.10	CEM III/A	5	75
19	1.4	0.32	0.15	CEM III/A	5	77
20	1.5	0.35	0.20	CEM III/A	3	74
21	1.3	0.41	0.00	CEM I + SF	2	58
22	1.4	0.29	0.05	CEM I + SF	9	64
23	1.5	0.32	0.10	CEM I + SF	9	64
24	1.1	0.35	0.15	CEM I + SF	13	54
25	1.2	0.38	0.20	CEM I + SF	8	58

**Table 6:** Air-entrainment and density of self-compacting mixture.

Series No.	*φ*	w/b	AEA	Cement Type	Air Content 5 min	Air Content 15 min	Difference in Air Content t = 5, t = 15	Density of Fresh Mixture
	-	-	% Mass of Cement [m.C]	-	%	%	s	kg/m^3^
1	1.2	0.32	0.00	CEM II/B-V	3.4	2.83	0.57	2268
2	1.3	0.35	0.05	CEM II/B-V	2.2	1.52	0.68	2225
3	1.4	0.38	0.10	CEM II/B-V	4.4	3.61	0.79	2147
4	1.5	0.41	0.15	CEM II/B-V	4	3.17	0.83	2130
5	1.1	0.29	0.20	CEM II/B-V	3.2	2.79	0.41	2183
6	1.4	0.35	0.00	CEM II/B-M	4.6	3.85	0.75	2262
7	1.5	0.38	0.05	CEM II/B-M	3.4	2.57	0.83	2290
8	1.1	0.41	0.10	CEM II/B-M	5.8	5.14	0.66	2200
9	1.2	0.29	0.15	CEM II/B-M	1.6	1.12	0.48	2180
10	1.3	0.32	0.20	CEM II/B-M	4.2	3.63	0.57	2229
11	1.5	0.29	0.00	CEM II/B-S	2	1.32	0.68	2185
12	1.1	0.32	0.05	CEM II/B-S	2.4	1.90	0.50	2042
13	1.2	0.35	0.10	CEM II/B-S	3	2.37	0.63	2132
14	1.3	0.38	0.15	CEM II/B-S	5.2	4.53	0.67	2182
15	1.4	0.41	0.20	CEM II/B-S	2	1.24	0.76	2192
16	1.2	0.41	0.05	CEM III/A	8.6	7.87	0.73	2185
17	1.1	0.38	0.00	CEM III/A	6.4	5.76	0.64	2138
18	1.3	0.29	0.10	CEM III/A	1.8	1.29	0.51	2223
19	1.4	0.32	0.15	CEM III/A	3.4	2.79	0.61	2225
20	1.5	0.35	0.20	CEM III/A	4.8	4.08	0.72	2153
21	1.3	0.41	0.00	CEM I + SF	8.6	7.80	0.80	2160
22	1.4	0.29	0.05	CEM I + SF	5.4	4.84	0.56	2262
23	1.5	0.32	0.10	CEM I + SF	6.6	5.92	0.68	2195
24	1.1	0.35	0.15	CEM I + SF	5	3.88	1.12	2220
25	1.2	0.38	0.20	CEM I + SF	6	5.36	0.64	2180

**Table 7:** Parameters of concrete porosity structure. According to EN 480-11 [32], *L*—pores distribution indicator, *α* applied designations—the specific surface of pores, *A*—air content in hardened concrete, *A*_300_—the content of micropores of the diameter below 300 μm.

Series No.	*φ*	w/b	AEA	Cement Type	*L*	*A*	*A*_300_	*α*
	-	-	% Mass of Cement [m.C]	-	[mm]	[%]	[%]	[mm^−1^]
1	1.2	0.32	0.00	CEM II/B-V	0.28	4.06	1.74	25.47
2	1.3	0.35	0.05	CEM II/B-V	0.42	2.09	0.5	17.86
3	1.4	0.38	0.10	CEM II/B-V	0.33	3.99	2.05	18.9
4	1.5	0.41	0.15	CEM II/B-V	0.31	4.26	1.96	21.24
5	1.1	0.29	0.20	CEM II/B-V	0.27	4.09	2.21	10.09
6	1.4	0.35	0.00	CEM II/B-M	0.42	2.06	0.8	17.37
7	1.5	0.38	0.05	CEM II/B-M	0.28	4.21	2.09	17.99
8	1.1	0.41	0.10	CEM II/B-M	0.28	6.57	2.73	21.15
9	1.2	0.29	0.15	CEM II/B-M	0.44	1.2	0.31	11.84
10	1.3	0.32	0.20	CEM II/B-M	0.21	5.04	1.97	21.93
11	1.5	0.29	0.00	CEM II/B-S	0.43	1.4	0.52	11.29
12	1.1	0.32	0.05	CEM II/B-S	0.31	3.69	1.3	20.98
13	1.2	0.35	0.10	CEM II/B-S	0.28	4.57	2.73	21.15
14	1.3	0.38	0.15	CEM II/B-S	0.23	6.18	1.62	17.48
15	1.4	0.41	0.20	CEM II/B-S	0.36	2.16	1.71	16.38
16	1.2	0.41	0.05	CEM III/A	0.15	8.51	3.2	28.17
17	1.1	0.38	0.00	CEM III/A	0.18	6.66	1.96	21.24
18	1.3	0.29	0.10	CEM III/A	0.33	2.77	1.45	18.9
19	1.4	0.32	0.15	CEM III/A	0.3	3.72	1.18	17.02
20	1.5	0.35	0.20	CEM III/A	0.28	5.34	1.45	22.83
21	1.3	0.41	0.00	CEM I + SF	0.13	8.59	2.1	36.14
22	1.4	0.29	0.05	CEM I + SF	0.32	5.23	0.95	24.91
23	1.5	0.32	0.10	CEM I + SF	0.23	7.3	1.18	24.24
24	1.1	0.35	0.15	CEM I + SF	0.31	5.82	0.91	16.07
25	1.2	0.38	0.20	CEM I + SF	0.26	6.49	2.99	19.04

**Table 8:** Derived indicators of early air retention and fresh-to-hardened air transfer calculated from Table 6 and Table 7.

No.	AEA % Mass of Cement [m.C]	*A*_5_ [%]	*A*_15_ [%]	Δ*A* [p.p.]	R_15_ [-]	Relative Loss [%]	*A*/*A*_15_ [-]	*A*_300_/*A* [%]
1	0.00	3.40	2.83	0.57	0.83	16.8	1.43	42.9
2	0.05	2.20	1.52	0.68	0.69	30.9	1.38	23.9
3	0.10	4.40	3.61	0.79	0.82	18.0	1.11	51.4
4	0.15	4.00	3.17	0.83	0.79	20.8	1.34	46.0
5	0.20	3.20	2.79	0.41	0.87	12.8	1.47	54.0
6	0.00	4.60	3.85	0.75	0.84	16.3	0.54	38.8
7	0.05	3.40	2.57	0.83	0.76	24.4	1.64	49.6
8	0.10	5.80	5.14	0.66	0.89	11.4	1.28	41.6
9	0.15	1.60	1.12	0.48	0.70	30.0	1.07	25.8
10	0.20	4.20	3.63	0.57	0.86	13.6	1.39	39.1
11	0.00	2.00	1.32	0.68	0.66	34.0	1.06	37.1
12	0.05	2.40	1.90	0.50	0.79	20.8	1.94	35.2
13	0.10	3.00	2.37	0.63	0.79	21.0	1.93	59.7
14	0.15	5.20	4.53	0.67	0.87	12.9	1.36	26.2
15	0.20	2.00	1.24	0.76	0.62	38.0	1.74	79.2
16	0.05	8.60	7.87	0.73	0.92	8.5	1.08	37.6
17	0.00	6.40	5.76	0.64	0.90	10.0	1.16	29.4
18	0.10	1.80	1.29	0.51	0.72	28.3	2.15	52.3
19	0.15	3.40	2.79	0.61	0.82	17.9	1.33	31.7
20	0.20	4.80	4.08	0.72	0.85	15.0	1.31	27.2
21	0.00	8.60	7.80	0.80	0.91	9.3	1.10	24.4
22	0.05	5.40	4.84	0.56	0.90	10.4	1.08	18.2
23	0.10	6.60	5.92	0.68	0.90	10.3	1.23	16.2
24	0.15	5.00	3.88	1.12	0.78	22.4	1.50	15.6
25	0.20	6.00	5.36	0.64	0.89	10.7	1.21	46.1

Note: *A*_5_ and *A*_15_ are fresh air contents after 5 and 15 min, respectively; Δ*A* = *A*_5_ − *A*_15_; R_15_ = *A*_15_/A_5_; *A*/*A*_15_ expresses fresh-to-hardened air transfer; *A*_300_/*A* expresses the share of micropores below 300 μm in the total hardened air content.

**Table 9:** ANOVA for fresh air content after 5 min, *A*_5_ [%].

Source	df	SS	MS	F	*p*-Value	Partial η^2^
AEA dosage	4	3.888	0.972	0.330	0.850	0.142
binder type	4	36.784	9.196	3.122	0.080	0.609
w/b	4	26.704	6.676	2.266	0.151	0.531
*φ*	4	1.296	0.324	0.110	0.976	0.052
Residual	8	23.568	2.946	-	-	-

Model R^2^ = 0.744; adjusted R^2^ = 0.233; Shapiro–Wilk *p* (residuals) = 0.791.

**Table 10:** ANOVA for absolute air loss between 5 and 15 min, ΔA [p.p.].

Source	df	SS	MS	F	*p*-Value	Partial η^2^
AEA dosage	4	0.0416	0.0104	0.718	0.603	0.264
binder type	4	0.0483	0.0121	0.833	0.540	0.294
w/b	4	0.2431	0.0608	4.190	0.040	0.677
*φ*	4	0.0541	0.0135	0.932	0.492	0.318
Residual	8	0.1160	0.0145	-	-	-

Model R^2^ = 0.769; adjusted R^2^ = 0.308; Shapiro–Wilk *p* (residuals) < 0.001.

**Table 11:** ANOVA for total hardened air content, *A* [%].

Source	df	SS	MS	F	*p*-Value	Partial η^2^
AEA dosage	4	1.711	0.428	0.154	0.956	0.072
binder type	4	37.058	9.265	3.345	0.069	0.626
w/b	4	30.007	7.502	2.708	0.107	0.575
*φ*	4	10.990	2.748	0.992	0.464	0.332
Residual	8	22.160	2.770	-	-	-

Model R^2^ = 0.783; adjusted R^2^ = 0.348; Shapiro–Wilk *p* (residuals) = 0.620.

**Table 12:** ANOVA for spacing factor, *L* [mm].

Source	df	SS	MS	F	*p*-Value	Partial η^2^
AEA dosage	4	0.0048	0.0012	0.219	0.920	0.099
binder type	4	0.0332	0.0083	1.524	0.283	0.432
w/b	4	0.0547	0.0137	2.508	0.125	0.556
*φ*	4	0.0223	0.0056	1.025	0.450	0.339
Residual	8	0.0436	0.0054	-	-	-

Model R^2^ = 0.725; adjusted R^2^ = 0.175; Shapiro–Wilk *p* (residuals) = 0.485.

**Table 13:** ANOVA for micropore content, *A*_300_ [%].

Source	df	SS	MS	F	*p*-Value	Partial η^2^
AEA dosage	4	2.869	0.717	1.937	0.198	0.492
binder type	4	0.254	0.064	0.172	0.947	0.079
w/b	4	6.012	1.503	4.058	0.044	0.670
*φ*	4	2.404	0.601	1.623	0.259	0.448
Residual	8	2.963	0.370	-	-	-

Model R^2^ = 0.796; adjusted R^2^ = 0.387; Shapiro–Wilk *p* (residuals) = 0.463.

**Table 14:** ANOVA for specific surface of air voids, *α* [mm^−1^].

Source	df	SS	MS	F	*p*-Value	Partial η^2^
AEA dosage	4	122.300	30.575	1.798	0.223	0.473
binder type	4	156.095	39.024	2.294	0.148	0.534
w/b	4	240.823	60.206	3.540	0.060	0.639
*φ*	4	65.694	16.423	0.966	0.476	0.326
Residual	8	136.069	17.009	-	-	-

Model R^2^ = 0.811; adjusted R^2^ = 0.434; Shapiro–Wilk *p* (residuals) = 0.809.

**Table 15:** Consolidated complete ANOVA outputs for the main response variables.

Response	Source	df	SS	MS	F	*p*-Value	Partial η^2^
*A*_5_ [%]	AEA dosage	4	3.888	0.972	0.330	0.850	0.142
*A*_5_ [%]	binder type	4	36.784	9.196	3.122	0.080	0.609
*A*_5_ [%]	w/b	4	26.704	6.676	2.266	0.151	0.531
*A*_5_ [%]	*φ*	4	1.296	0.324	0.110	0.976	0.052
*A*_5_ [%]	Residual	8	23.568	2.946	-	-	-
Δ*A* [p.p.]	AEA dosage	4	0.0416	0.0104	0.718	0.603	0.264
Δ*A* [p.p.]	binder type	4	0.0483	0.0121	0.833	0.540	0.294
Δ*A* [p.p.]	w/b	4	0.2431	0.0608	4.190	0.040	0.677
Δ*A* [p.p.]	*φ*	4	0.0541	0.0135	0.932	0.492	0.318
Δ*A* [p.p.]	Residual	8	0.1160	0.0145	-	-	-
*A* [%]	AEA dosage	4	1.711	0.428	0.154	0.956	0.072
*A* [%]	binder type	4	37.058	9.265	3.345	0.069	0.626
*A* [%]	w/b	4	30.007	7.502	2.708	0.107	0.575
*A* [%]	*φ*	4	10.990	2.748	0.992	0.464	0.332
*A* [%]	Residual	8	22.160	2.770	-	-	-
*L* [mm]	AEA dosage	4	0.0048	0.0012	0.219	0.920	0.099
*L* [mm]	binder type	4	0.0332	0.0083	1.524	0.283	0.432
*L* [mm]	w/b	4	0.0547	0.0137	2.508	0.125	0.556
*L* [mm]	*φ*	4	0.0223	0.0056	1.025	0.450	0.339
*L* [mm]	Residual	8	0.0436	0.0054	-	-	-
*A*_300_ [%]	AEA dosage	4	2.869	0.717	1.937	0.198	0.492
*A*_300_ [%]	binder type	4	0.254	0.064	0.172	0.947	0.079
*A*_300_ [%]	w/b	4	6.012	1.503	4.058	0.044	0.670
*A*_300_ [%]	φ	4	2.404	0.601	1.623	0.259	0.448
*A*_300_ [%]	Residual	8	2.963	0.370	-	-	-
*α* [mm^−1^]	AEA dosage	4	122.300	30.575	1.798	0.223	0.473
*α* [mm^−1^]	binder type	4	156.095	39.024	2.294	0.148	0.534
*α* [mm^−1^]	w/b	4	240.823	60.206	3.540	0.060	0.639
*α* [mm^−1^]	*φ*	4	65.694	16.423	0.966	0.476	0.326
*α* [mm^−1^]	Residual	8	136.069	17.009	-	-	-

Note: The table reports main effects calculated within the Graeco-Latin square screening design. Residual df = 8 for each response; partial η^2^ was calculated as SS_effect/(SS_effect + SS_residual).

**Table 16:** Summary interpretation of ANOVA results for the analysed response variables.

Response Variable	Table	Significant Factor(s), *p* < 0.05	Near-Threshold Trend(s), 0.05 ≤ *p* < 0.10	Interpretation
Initial fresh air content *A*_5_	Table 9	None	binder type, *p* = 0.080	Fresh air content was system-dependent; AEA alone was not confirmed as a main effect.
Early air loss Δ*A*	Table 10	w/b, *p* = 0.040	None	Significant but interpreted cautiously because residual normality was not satisfied.
Hardened air content *A*	Table 11	None	binder type, *p* = 0.069	Fresh-to-hardened correlation was strong; individual main factors were not universal controls.
Spacing factor *L*	Table 12	None	None	Observed L variation requires combined pore-structure interpretation; φ is mechanistic, not statistically confirmed here.
Micropore content *A*_300_	Table 13	w/b, *p* = 0.044	None	Pore refinement was linked with paste-related conditions in the adopted design.
Specific surface *α*	Table 14	None	w/b, *p* = 0.060	Near-threshold tendency only; no confirmed main effect at α = 0.05.

## Data Availability

The original contributions presented in this study are included in the article. Further inquiries can be directed to the corresponding author.
